# Stimulus and Network Dynamics Collide in a Ratiometric Model of the Antennal Lobe Macroglomerular Complex

**DOI:** 10.1371/journal.pone.0029602

**Published:** 2012-01-10

**Authors:** Kwok Ying Chong, Alberto Capurro, Salah Karout, Timothy Charles Pearce

**Affiliations:** Centre for Bioengineering, Department of Engineering, University of Leicester, Leicester, United Kingdom; The University of Plymouth, United Kingdom

## Abstract

Time is considered to be an important encoding dimension in olfaction, as neural populations generate odour-specific spatiotemporal responses to constant stimuli. However, during pheromone mediated anemotactic search insects must discriminate specific ratios of blend components from rapidly time varying input. The dynamics intrinsic to olfactory processing and those of naturalistic stimuli can therefore potentially collide, thereby confounding ratiometric information. In this paper we use a computational model of the macroglomerular complex of the insect antennal lobe to study the impact on ratiometric information of this potential collision between network and stimulus dynamics. We show that the model exhibits two different dynamical regimes depending upon the connectivity pattern between inhibitory interneurons (that we refer to as fixed point attractor and limit cycle attractor), which both generate ratio-specific trajectories in the projection neuron output population that are reminiscent of temporal patterning and periodic hyperpolarisation observed in olfactory antennal lobe neurons. We compare the performance of the two corresponding population codes for reporting ratiometric blend information to higher centres of the insect brain. Our key finding is that whilst the dynamically rich limit cycle attractor spatiotemporal code is faster and more efficient in transmitting blend information under certain conditions it is also more prone to interference between network and stimulus dynamics, thus degrading ratiometric information under naturalistic input conditions. Our results suggest that rich intrinsically generated network dynamics can provide a powerful means of encoding multidimensional stimuli with high accuracy and efficiency, but only when isolated from stimulus dynamics. This interference between temporal dynamics of the stimulus and temporal patterns of neural activity constitutes a real challenge that must be successfully solved by the nervous system when faced with naturalistic input.

## Introduction

The macroglomerular complex (MGC) is a structure within the antennal lobe (AL) of some insect species specialized to process pheromone information. It is composed of a group of specialized glomeruli located where the antennal nerve first enters the AL and is almost entirely functionally separated from the general olfactory system. As such, the MGC can be considered to be an AL in miniature, but with the very specific task of identifying the presence of one behaviourally significant chemical cue: the species-specific pheromone blend [1]. This system is very important for reproduction. During anemotactic orientation for seeking a mate, detecting and identifying the correct ratio of sex pheromone components of a calling female is vital for enabling male moths to fly up the pheromone plume to locate the female [2]–[4].

Behavioural evidence shows that male moths prefer the full pheromone blend extracted directly from female glands [5]. In wind tunnel experiments, male oriental fruit moths, *Grapholita molesta*, can distinguish between the full pheromone blend and chemical cues composed of incomplete blends of major pheromone component agonists [5]. This moth will usually complete anemotaxis to the source of the full pheromone extract, even when another plume is simultaneously presented composed of an incomplete blend. Also, there are examples of species of moths that live in overlapping habitats that use common pheromone components but do not interbreed because males are attracted only to the conspecific pheromone blend [6].

The olfactory receptor neurons (ORNs) responsible for relaying pheromone information to the MGC are very specifically tuned to individual pheromone components [7]. Each component is detected by just one receptor type that sends convergent axons to the projection neurons (PNs) of just one glomerulus, while the local neurons (LNs) innervate many glomeruli [8], [9]. Thus, information regarding each component is integrated in a different glomerulus. This makes the MGC ideal for modelling since it offers a relatively simple system with a specific task.

Electrophysiological studies have found MGC neurons that respond to all pheromone stimuli or selectively to just one pheromone component, or even to a particular blend or ratio [10]–[14]. For *S. littoralis* in particular, some interneurons responded to one, two, three or all four pheromone compounds, and some just to the mixture of all four indicating that MGC second order neurons are involved in pheromone blend encoding.

The behavioural and electrophysiological studies mentioned above indicate that moths can detect ratios of pheromone blends very precisely. This is a difficult task because it requires the detection of both the individual components and their relative concentrations when presented as a blend varying in time. The optimal solution requires non-overlapping information channels as described in the MGC, and its associated ORN tunings. Since the male moth needs to detect pheromone ratios in odour plumes, the possible conflict between the temporal dynamics of the stimulus with the temporal patterns of neural activity that are used to encode concentration ratios constitutes a real challenge that is successfully solved by the insect in behavioural situations. In this context, we present here a neural network model that is able to encode ratios between the concentrations of two odorants in a blend, and investigate how this goal can be achieved using two types of neural population dynamics that are associated with two different encoding strategies. In particular, we focus on the performance of these two different dynamics to detect concentration ratios when the stimulus pattern displays complex variations in time as happens in odour plumes.

In our computational model [15], the connections are biologically constrained to known morphological details of moth MGC. Two types of dynamics are generated with networks that share the same neural connectivity, except for the LN-to-LN inhibitory connections, that we call fixed point attractor (FPA) and limit cycle attractor (LCA) behaviours, reminiscent of “Winner-Takes-All” and “Winnerless Competition”, respectively [16]. FPA networks employ symmetrical inhibition between competitive elements, and the resulting neural network encodes stimulus through the spatial identity of neurons, while LCA networks have asymmetrical inhibition and the output is rich in spatiotemporal dynamics. We compare the ability of the two encoding schemes to represent binary odour ratios and in the process provide insights about certain details of the network connectivity that are still unknown.

## Methods

### Modelling of Neurons

Individual neuron dynamics are modelled using first order differential equation that describes the evolution of the firing-rate activity over time,
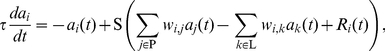
(1)where 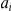 is the activation level of the 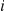
th interneuron; 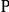 is the subset of neurons that are PNs, and 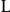 the subset of neurons that are LNs; 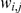 is the strength of synaptic influence of 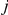 on the activity of 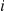 (similarly for 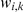); 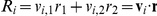 is the afferent input from receptor neurons to the 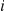
th interneuron, which is the dot product of glomerular inputs from the two receptor neuron types, 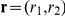, weighted by the strength of connections, 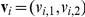; 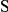 is a sigmoidal squashing function; and 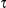 (set at 10 ms for all PNs and at 20 ms for all LNs) is the time constant governing the speed of neuronal dynamics. 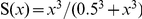 for 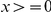, and 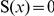 for 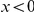, is a rectified sigmoid function that limits the neuronal activity to values between 0 and 1 while still allowing a linear-like response to a range of input levels between non-activation and saturation. Note that synaptic influence from PNs, 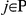, is excitatory and therefore positive, while LNs, 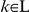, are inhibitory and negative. More details for the determination of the connection weights 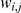 and 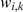 are given in the next section. Simulations were carried out on a PC running MATLAB using customized code. The evolution of the neuronal firing-rates over time was calculated using integration by a Runge-Kutta algorithm with fixed time-steps of 1 ms. A Gaussian noise 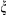 (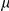 = 0, 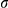 = 5
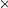
10–4) was added to this equation at each time-step to create non-deterministic firing-rates. The value of 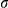 was chosen such that the neuronal activity was not completely dominated by noise, but still generated variability between repetitions to allow a comparison of the robustness to noise between different instances of the model.

The initial activation values were taken from a Gaussian random distribution with 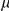 = 0.01 and 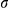 = 0.0025. In both LCA and FPA models, the values of 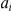 rapidly converged to an equilibrium point that was zero in the absence of stimulation (i.e., there was no spontaneous activity). We waited 100 ms from the start of the simulation until the stimulus onset to ensure that the initial conditions did not influence the comparison between both dynamics (FPA and LCA).

### Network Connectivity

**Figure 1:**
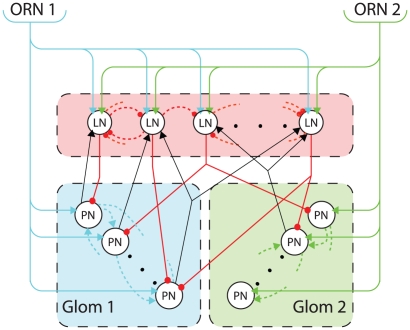
Neuronal connectivity schematic. Excitatory connections are represented with arrows and inhibitory connections with circles. Just two glomeruli are modelled, which are the convergent sites for axons of two ORN types (cyan for ORN1 and light green for ORN2). The receptor neurons provide afferent excitation to both classes of AL neurons. PNs, being exclusively uniglomerular, receive excitation from just one type of receptor, while LNs, being multiglomerular, receive excitation from multiple receptor types. LNs and PNs have mutual random connections (red and black solid lines – see Methods). PNs innervating a single glomerulus form random excitatory interconnections with fixed probability (dashed lines – see Methods). Two cases of inhibitory LN interconnectivity are considered: random connections of fixed probability to produce LCA behaviour (as in the example shown here) and all-to-all connectivity generating FPA behaviour (not shown).

The general connectivity of the network (Figure 1) was set according to morphological studies of the moth MGC [8], [9], [13], [17]–[21]. The number of MGC glomeruli equals the number of behaviourally relevant pheromone components, with each ORN type projecting to one MGC glomerulus [21]. This number can typically range between 1 and 8, depending on the species of moth. Since the encoding of blend ratio is investigated here, a two-component pheromone blend and, accordingly, two glomeruli are simulated.

The number of PNs and LNs has large variation in different species of moth. In *Manduca sexta* there are 35–40 PNs in the MCG, and the total number of LNs is 360 in the whole AL (Table 1 in [17]). From this number of LNs the proportion that belongs to the MGC is undetermined, but it is likely to be only a minor fraction because most glomeruli are part of the general olfaction system. In this context, we decided to use 30 PNs (divided in 2 glomeruli) and 30 multiglomerular LNs as a plausible approximation that allows a rich dynamic behaviour in our model network.

In the moth MGC, pheromone sensitive ORNs are specifically tuned to just one pheromone component and relay information to the PNs of one particular glomerulus [9], while the connectivity within PNs is mostly uniglomerular [19]. The input to each glomerulus was taken to be the aggregated activity of ORNs activated by the respective pheromone component, represented by a scalar value within a two element vector 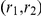. Therefore, each PN in the model receives input from just one receptor type (either 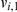 or 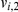 = 0, for all 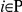) via a randomly weighted connection (in order to give most PNs direct afferent excitation, weights were drawn from a normal distribution, 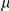 = 1, 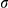 = 1, with negative values rectified to 0), and has random excitatory interconnections with other PNs of the same glomerulus. PNs have 0.8 probability of synapsing to another PN within a glomerulus, with a relatively small connection weight of 0.0125
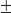
0.1 (negative values were rectified to 0). These values have been chosen so as to not cause runaway excitation (PN trajectories showed little change for weight values between 0 and 0.4 in FPA and from 0 to 1.6 in LCA).

In contrast to PNs, the LNs of the moth MGC are generally multiglomerular and receive input from many types of pheromone sensitive ORNs [13]. Therefore, in the model LNs receive input from both receptor types, so receiving information for both pheromone components. The connection probability was set to 1.0, with weights drawn from a normal distribution, 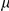 = 1, 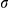 = 1, with negative values rectified to 0.

As output and input synapses have been identified between LNs and PNs in many species [13], these contacts were included in our model. Inhibitory connections from the LNs to the PNs were chosen randomly. A LN had a probability of 0.2 to connect to any PN, irrespective of the glomerulus to which the PN is associated, and a high strength weighting for that connection (−2.5
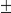
0.1). The high strength of connection ensured that any post-synaptic PN would be completely inhibited, and as such, any value smaller than −1 would suffice. Relatively sparse connectivity from LNs to PNs was required to ensure that not all PNs were completely inhibited during odour presentation. Although the exact value was not important, 0.2 provided a balance that allowed LNs to influence but not overwhelm PN response. We also included excitatory feedback from PNs to LNs with a connection probability of 0.5, and synaptic weight set to 0.033
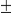
0.1 (negative values were rectified to 0).

FPA network and LCA network models differed by how interconnections between LNs were determined. For FPA networks, competition between cells was created through mutual inhibition, so LNs were given all-to-all connections with a high inhibitory connection strength (−15
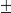
0.1). For LCA networks, cells were connected asymmetrically with high inhibitory connection strength (−15
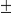
0.1), leading to uneven competition that prevents a long-term winner, and therefore prevents a stable equilibrium point. To generate this asymmetry, connections between LNs were chosen randomly with a probability of 0.25 for any LN connecting to any other. For this value, the subnetworks of LNs generate switching behaviour. Indeed, any value between 0.2 and 0.4 could also generate switching behaviour. However, for values less than this the subnetworks would lack LN interactions, and for values greater, subnetworks would become largely symmetrical. In either case switching behaviour would not occur.

In FPA models, for each LN that contacts another there exists a reciprocal connection (with both synaptic weights being drawn from the same random distribution), so the coupling must be symmetric. When the connection probability turns lower (e.g., 0.25 in LCA), it becomes very probable that if a given LN contacts another the opposite may not be true, giving rise to an asymmetric connectivity pattern in the LN subnetwork.

20 realizations of each network type (LCA and FPA) were created following the above rules so as to investigate the general properties of the model and the effects of the noise level. The connectivity is summarized in Figure 1.

## Results

### Network Behaviours

**Figure 2:**
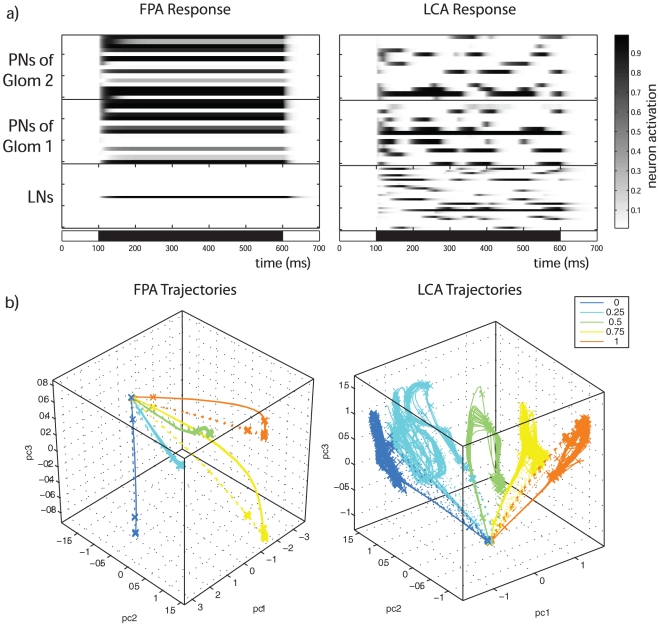
Examples of modelled responses. a) These two panels show examples of the response to stimulation from a FPA network (left) and a LCA network (right). The bar in the horizontal abscissa indicates stimulus duration and timing. For illustrative purposes, a 0.5∶0.5 ratio stimulus was used. In the sub-panels, the rows are the activity for each neuron. The top two sub-panels show PN activity in the two glomeruli, while the bottom sub-panel shows LN activity. The FPA model displays transient activity patterns before settling to a stable spatial pattern around 100 ms after stimulus onset, whereas the LCA model displays rich temporal patterning during the stimulus period. b) Trajectories of the PN responses to different input ratios for the same model realizations displayed in the rasters above, each with 20 repeats perturbed with different realizations of Gaussian white noise of 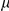 = 0 and 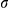 = 5
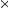
10–4. The first three principal components have been taken to produce 3-dimensional plots so that a point in these plots represents a spatial response pattern at an instant in time (bin size was 5 ms). Solid lines indicate the trajectories during stimulation, while dotted lines indicate return trajectories after stimulation. The crosses are plotted at regular time intervals of 50 ms, and thus indicate the local velocity of the trajectories and its variability between trials. The different network behaviours are also apparent in these plots. The time in which the mean Euclidean distance between the trajectories reaches a maximum value is 358
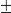
198 ms for FPA, and 226
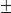
143 ms for LCA (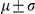 for 20 model realizations, p = 0.02 in the t-test).

The behaviours of the MGC models created are illustrated by an example response of a FPA network, and one of a LCA network (left and right panels in Figure 2a, respectively). The LNs in the FPA network compete directly with one another through the all-to-all inhibitory connectivity. The random ORN to LN connectivity determines that for any ratio there is one LN that receives greater excitation, and inhibits its competitors becoming dominant. In the left panel of Figure 2a, this FPA competition can be seen in the LN responses: after a short initial transient only one LN remains active, and the network settles into a stable spatial pattern of activity. This is also reflected in the PN responses, showing a very brief activity transient immediately after stimulus onset until a stable pattern is established. In contrast, the LNs in the LCA network do not settle to a stable spatial pattern within the time period considered (500 ms), but change continuously, driving the PN activity to do the same. This results in a sequence of PN activation patterns comprising rich spatiotemporal dynamics (right panel in Figure 2a). We investigated the evolution of these complex dynamics for longer stimulus durations (5 seconds) and found that the network typically reaches a fixed point (Figure S1) or a periodic orbit (not shown) after 0.5 to 1 second of simulation. This later fixed point includes many LNs active at the same time, in contrast with the earlier fixed point of the FPA network (Figure 2a) in which only a single LN remains active. As pheromone information in natural odour plumes is presented in pulses of short duration, this fixed point is unlikely to be reached in the moth during behaviuoral situations. For this reason we kept the term LCA, as it refers to the dynamics observed during the stimulus period used in our simulations (500 ms). However, this issue is interesting from a theoretical point of view in the comparison between LCA and “Winerless Competition” types of neuronal dynamics because this late fixed point is not reached in the last case, although it was observed in electrophysiological recordings performed in the locust AL [22] (see Discussion).

The transient PN responses can also be seen in the trajectory plots depicted in the lower panels of Figure 2. The FPA model responses (left panel in Figure 2b) show fast onset transients that lead to stable attractors at a given instant. The system stays near these stable attractors until the return transients after stimulation. The LCA model (right panel in Figure 2b) also displays fast transients from rest at stimulus onset, and then enters into complex spatio-temporal patterns which are both sensitive to the input ratio and repeatable over trials perturbed with different realizations of noise.

The trajectories show how the responses differ across input ratios in each network (lower panels of Figure 2). In both networks, the PN population responses to different ratios are separated. In the FPA model, trajectories never cross for different ratios during the transient phase or as these approach the fixed point. The separation of the fixed points for different ratios indicate reliable encoding of the blend ratios considered over time. In the LCA model, the trajectories are shown to be localized in different regions of the PN phase space that do not overlap. In both networks, the trajectories also show that responses that are closest together are induced by neighbouring ratios, suggesting that the response trajectories and their associated attractors vary smoothly and continuously with changes in the input ratio.

### Conflict between Stimulus Dynamics and Network Dynamics

An issue with using time as an encoding dimension for odour quality is that this temporally structured code may become confounded with the dynamics of the stimulus. This is especially problematic for animals such as the moth that perform anemotactic orientation in odour plumes, where the stimulus dynamics can be very complex and have been shown to be behaviourally relevant [23], [24].

**Figure 3:**
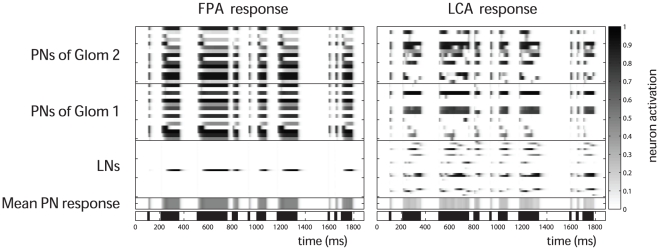
Responses to randomly timed stimulus pulses. Responses to randomly timed stimulus pulses. The FPA network (left) and the LCA network (right) were presented with the same randomly timed stimulus. Both models were able to follow the odour pulses, and at each onset of stimulus, the initial transients governed by networks dynamics appear to start anew. The averaged PN activity shows that both models follow the odour pulses closely and this averaged activity accurately relays the timing information of the stimulus.

Both FPA and LCA models can preserve and accurately pass on the stimulus dynamics to downstream neural processes, as illustrated in Figure 3. The smallest pulse in Figure 3 is around 40 ms, which is realistic for odour plumes [24]. However, we wondered if the stimulus dynamics reflected in the activity of the PN population could interfere with the encoding of the blend ratio. To assess this issue, we correlated the spatiotemporal output patterns of the PN population for a single stimulus pulse of long duration with the patterns obtained from stimulation with differently timed pulses. This result is presented in the next section for both the FPA and LCA models. The spatiotemporal output was taken to be the activity of the PNs partitioned into time-bins. The mean activity for each PN was found over each time-bin, 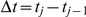 = 10 ms, for each time-step 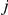. This produced a matrix, 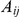, composed of a time series of activity for each PN, 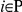:
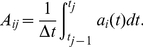
(2)


#### Correlating PN Population Responses

To assess the evolution of the PN code over time and its dependence upon the stimulus, the matrix of spatiotemporal responses to a 500 ms duration stimulus, 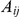, was broken into a set of column vectors 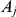, each describing the instantaneous spatial activation pattern of the PNs at the 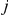
th time-bin. This process was repeated for the PN responses to a differently timed pulsed stimulus, generating the set of vectors 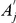. The similarity between PN responses for the two different stimuli and at different times 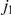 and 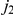 was measured using the correlation coefficient between response vectors defined as
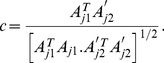
(3)


**Figure 4:**
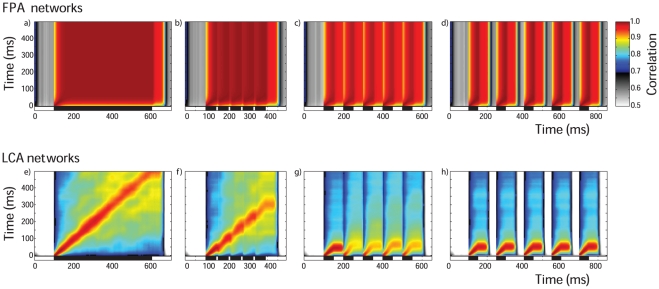
Cross correlation and stimulus timing. Cross correlation and stimulus timing. Time-binning the PN population responses results in a series of instantaneous spatial activity patterns. The spatial activity patterns for a 500 ms duration stimulus (vertical axis) were correlated with the spatial patterns from differently timed pulsed stimuli (indicated by bars on horizontal abscissas). This was done for FPA networks (a–d) as well as for LCA networks (e–h). The pulse patterns are: a) one 500 ms pulse and; b) five 50 ms pulses with inter-stimulus intervals of 10 ms, c) 50 ms and d) 100 ms. The same patterns were used for LCA (e–h). The correlation coefficients shown are mean values taken from 400 random input ratios and 20 networks. The different network behaviours between FPA and LCA networks result in very different looking cross correlation maps for the 500 ms pulse results (a and e). FPA networks show high correlation throughout periods of stimulation, indicating that the spatial patterns are very similar across time-bins (a). LCA networks display lower correlations, with highest values in a thin region along the diagonal (e). For both FPA and LCA networks, very short inter-stimulus intervals of 10 ms have little effect except to slow the progression of the sequence of spatial patterns (b and f). The pulses with long inter-stimulus intervals of 100 ms do not interfere, and each pulse elicits a separated response that is almost identical to each other pulse (d and h). However, for 50 ms inter-stimulus intervals, the tail of the previous pulse overlaps with the next response (c and g). This has no apparent effect on FPA models, but LCA models show a marked difference in the shape of pattern of correlation for pulses following the first.

In this way, we assessed the effect of inter-pulse intervals on the correlation (Figure 4).

The different network behaviours are clearly evident in the cross correlation diagrams. The stable spatial patterns of the FPA models result in high correlation coefficients between all time-steps for the 500 ms single pulse (Figure 4a). The large square region of almost perfect correlation shows that the network dynamics were relatively consistent over time. The transients leading to the stable attractor can be seen as a small region of high correlation on the diagonal immediately after stimulus onset. In contrast, the temporally rich behaviour of the LCA models results in relatively low correlation except for a narrow band on the upward diagonal (Figure 4e). This narrow diagonal shows that the changes in spatial patterns happen smoothly but quickly, with the width being no greater than 50 ms, as the spatial pattern must be switching within this time.

When 10 ms inter-stimulus intervals are introduced to break up the single long pulse, in both the FPA and LCA models (Figure 4b and f), the systems begin to return to rest at each interval, but these return transients have not enough time to get established. Thus, the progression of responses is temporarily halted at each interval, but then resumes at the onset of the next pulse.

Longer inter-stimulus intervals of 100 ms (Figure 4d and h) allows enough time for these networks to fully reset and start responses afresh, and so each pulse produces the same initial spatiotemporal response. For FPA models, there is still the block of high correlation for each pulse (Figure 4d), and for LCA models, there is the same diagonal for each pulse (Figure 4h). For both networks, the correlation pattern during the pulse is the same as the first 50 ms of the 500 ms pulse. In the next 50 ms after each pulse, the correlation pattern does not change significantly from that at stimulus offset. This indicates that the spatial response pattern does not change during the return transients. However, it decreases in strength until fading away due to the decorrelation introduced by the noise in the firing-rate.

In the case of 50 ms inter-stimulus intervals, this intermediate time length allows overlap between the return transients after each pulse and the initializing transients of the next pulse (Figure 4c and g). Given the simple temporal structure of the responses in the FPA models, the high correlation for each pulse is re-stablished, as happened for the 100 ms interval pulses (Figure 4c). However for LCA networks, 50 ms intervals do not allow a full reset in the dynamics as for longer intervals, nor a continuation of the temporal sequence as for shorter intervals (Figure 4g). Instead, except for the first 20 ms after stimulus onset, the spatial patterns appear to be altered and are no longer correlated with spatiotemporal response for the 500 ms pulse. This has a profound effect on the encoding of ratio identity by the spatiotemporal PN responses when the network is exposed to stimulus dynamics on certain time scales, as we show in the next section.

#### Identifying Ratios from PN Responses

In order to investigate how well FPA and LCA models encode odour ratios, we assessed the separability of the spatiotemporal outputs between ratios, and its robustness to noise. This was done by discriminant analysis, to quantify how well the input ratio could be identified from the PN responses. Stimuli were created by randomly selecting ratios from a uniform distribution. They were categorized into five ratio groups 0∶1, 0.25∶0.75, 0.5∶0.5, 0.75∶0.25, 1∶0 corresponding to 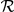 = 0, 0.25, 0.5, 0.75, 1 (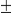
0.125) respectively. More formally, for a ratio 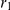
∶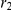, 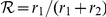.

**Figure 5:**
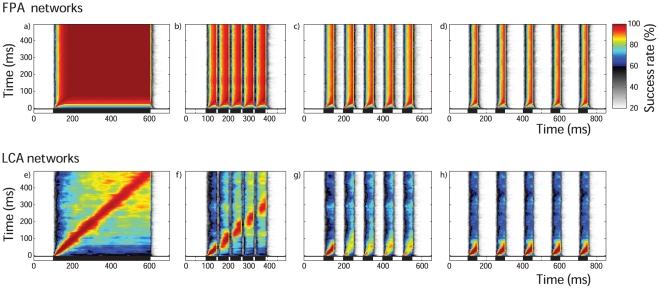
Cross classification and stimulus timing. Again, we use the series of instantaneous spatial patterns as in Figure 4. FPA (a–d) and LCA (e–h) networks were repeatedly stimulated with 500 ms pulses of five different ratios. The subsequent PN spatial patterns for each time-bin were used to train a linear model. The networks were also stimulated with the differently timed pulses of the same 5 ratios, generating spatial patterns which were then classified by the linear model, estimating which ratios generated each spatial pattern (see methods). The colour scale is the same for all panels, and shows the mean success rate for multiple trials and networks. 20% is the chance success rate. For the single pulse of 500 ms, there is high accuracy along the diagonal for both FPA and LCA networks (a and e). LCA networks have a thin region around the diagonal of high accuracy (e), indicating a smooth transition between spatial patterns, which are constantly switching. FPA networks simply show a block of high accuracy (a), indicating that the spatial patterns change very slowly or not at all. The dynamics is not affected for inter pulse intervals of 10 ms (b and f) and 100 ms (d and h). For inter pulse intervals of 50 ms, LCA models suffer a big drop in classification accuracy as the tail of responses to the first pulse is confounded with the spatiotemporal responses of subsequent pulses (g), while FPA responses allow classification in each pulse just as if they were presented individually (c).

In the same way as for correlation analyses, the spatiotemporal output was taken to be the time-binned PN responses. Thus, for each odour presentation, a time series of instantaneous PN activity patterns was constructed. A 500 ms pulse was used to generate a training set of 100 stimuli, which gave 100 instantaneous PN responses for each time-step. These time-steps were set on the vertical axis of the plots in Figure 5. Then, the time steps of a data set composed of 400 stimulations were placed on the horizontal axis and classified using discriminant analysis. In this way, we can visualize the times at which the data set is coherent with the training set and therefore allow correct classification. The colour code represents success rate of ratio blends correctly classified.

As part of the discriminant analysis algorithm, the covariance matrix of the variables has to be inverted. In this situation, redundant linearly dependent dimensions of the training set can cause failures in the calculations. To avoid this problem, principal component analysis (PCA) was applied beforehand to retain only the principal dimensions reflecting 90% of variance before classification was attempted.

Figure 5 confirms the underlying behaviours of the FPA and LCA models observed in Figure 4. FPA models show a large square region of almost perfect classification from 50 ms after stimulus onset to the stimulus end in both axes (Figure 5a). This is indicative of the constant spatial code generated by the stable attractor that the FPA networks attain after initial transients. The transients leading to the stable attractor are evident in the line of high classification rates along the diagonal in the first 50 ms after stimulus onset.

LCA models show a narrow band where the accuracy is greater than 90% (Figure 5e). The narrow width indicates the fast switching behaviour of the spatiotemporal PN code. This diagonal is pronounced for the whole stimulus duration in this analysis, showing that the temporal code spans the whole stimulation. There are also elevated success rates that are off of the diagonals during the last half of the stimulation. This is evidence of the LCA models entering limit cycles, repeating spatiotemporal output. However, these regions do not seem well defined since the properties of these limit cycles are different for each model instantiation and stimulus ratio. These features were also evident in the correlation analysis, but the colour scale of the diagram made it more difficult to visualize, and also the correlation analysis was more affected by the variability of the neural activity, which accumulates over time, gradually weakening the correlation.

The effect of short 10 ms intervals can be seen to momentarily halt the progression of the response dynamics, which resume at the next pulse (Figure 5b and f). During these intervals the drop in classification rates can be seen clearly. The 100 ms intervals provide enough separation for each pulse to start a completely new response for both FPA and LCA models (Figure 5d and h). The most interesting case of the intermediate length 50 ms intervals shows that FPA responses allow classification in each pulse just as if they were presented individually (Figure 5c). However, LCA models suffer a big drop in classification accuracy as the tail of responses to the first pulse is confounded with the spatiotemporal responses of subsequent pulses (Figure 5g). This shows that the spatiotemporal encoding scheme cannot reliably convey odour identity for certain dynamical stimuli, specifically when the stimulus dynamics are on a similar time scale thus interfering with the intrinsic dynamics of the LCA network.

### Time as an Encoding Dimension

#### Measure of Ratio Specificity

The advantage of using time as an extra encoding dimension is that, theoretically, it can greatly increase the potential encoding space available. No longer is the information confined to just a static spatial activity pattern, but can entail a sequence of spatial patterns, multiplying the possible representations with each time step. Here, we compare the spatiotemporal PN codes for the FPA and LCA models to investigate if the more temporally complex output of LCA models utilizes this advantage.

In this section, analyses are performed on the spatiotemporal code including time, not just instantaneous ‘snapshots’ as before. Here, the response to stimulation is taken to be a vector produced by all the elements of the matrix 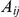 (as defined by equation 2), giving
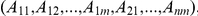
if 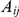 is an 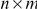 matrix. This makes a vector space with a dimension for each time-step for each PN.

**Figure 6:**
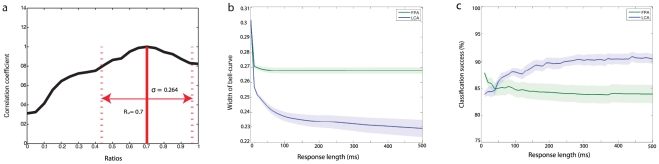
Ratio specificity, output reliability and code length. a) An example illustrating the bell-shaped correlation curve. Correlating the PN population response for a particular ratio, 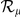, against all other ratios gives this approximately bell-shaped curve, which peaks at 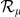 with a correlation coefficient of value 1. The more specific the PN response to a ratio, the sharper and thinner the bell-shaped curve. Thus, the width of the bell-curve, 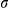, marked by the dotted lines (see text for definition) indicates the specificity of the PN response. b) The effect of response length on code specificity. This shows the specificity of the spatiotemporal PN responses as the response length taken into account is changed, indicating how the ability of the models to encode ratios is dependent on the temporal dynamics. The shaded areas show standard error. c) The effect of response length on reliability. Discriminant analysis was used to predict the input ratios from PN responses according a training set of responses (see text). The accuracy of this classification indicates the reliability of the PN responses for the identification of input ratios. This test was applied to different response lengths as in b). The accuracy of both types of networks becomes stable by 100 ms, with FPA networks at around 85% and LCA networks at around 91%. LCA networks start with lower accuracy but quickly increase and exceed that of FPA networks. The FPA networks' ability to hold ratio information actually decreases as they reach the stable attractors. Again, shaded areas represent the standard error.

When the response to one input ratio is correlated with other ratios, the closest ratios have highly correlating neuron responses while very different ratios have low correlating responses. This creates a bell-shaped curve (Figure 6a). We use the second moment about the peak correlation as a measure for the width of this bell-shaped curve, and thus the specificity of the PN code. Let the input ratio be denoted by the proportion of the input composed of type 1 receptor input, 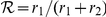, then the curve width, 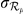, for a particular ratio, 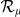, is defined to be
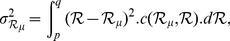
(4)where 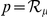 and 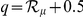 for 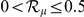, and 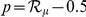 and 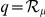 for 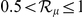 in order to avoid problems at the boundaries of 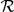. 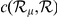 is the correlation coefficient for the spatiotemporal responses to the two ratios. The average width for a network was taken to be the mean width over ratios.

This bell-curve width is indicative of the specificity of the PN output to ratios. Since the correlation between the spatiotemporal output to different stimuli gives a measure for the similarity of the these outputs, a narrow bell-curve would be produced by a spatiotemporal code that changes greatly across ratios. The sharper the bell-curve, the better the differentiation can be of the input ratios from the spatiotemporal output. This measure for ratio specificity was used to assess the importance of time to FPA and LCA models. The time-length from stimulus onset of the spatiotemporal PN responses was changed, and the analyses were carried out for each time-length. In this way, we tested the effect of the time-length of the code on the ability of PN population responses to encode ratios.

The temporal consistency of the PN responses from FPA models determines that the ratio specificity is not enhanced by taking longer time-lengths of PN responses. The fixed spatial response patterns do not allow any more information to be conveyed once the transient patterns have completed. Therefore for FPA models, one would not expect that ratio specificity is dependent on code length once the stable attractor has been reached, and this is indeed the case (Figure 6b). Interestingly however, specificity is not dependent on code length, except for the first 10 ms transient phase before a stable spatial pattern is fully established, as depicted in Figure 6b by a horizontal plot for code lengths of over 50 ms.

In contrast, LCA models have much improved ratio specificity as time allowed for encoding is increased. The largest drop in bell-curve width is within the first 200 ms, after which the rate of change is reduced, and a stable value is reached by 500 ms (Figure 6b). This drop in bell-curve width corresponding to an increase in ratio specificity of the PN code demonstrates that the spatiotemporal activity of PN responses in LCA models does indeed utilize time as a coding dimension. Part of this effect can be related to the fact that PN responses are sparser because they receive larger inhibition from the LN population, which is more active due to a lesser degree of inhibitory self coupling.

In summary, our results suggest that a spatiotemporal code can and does carry more information than spatial-only code, allowing a more precise determination of the input ratio over time.

#### Response Length and Reliability

In this final section, we take the idea of the spatiotemporal code over limited time-lengths presented in Figure 6a, and apply it to the training and data set responses used in Figure 5. Then, we perform the discriminant analysis on this spatiotemporal output (Figure 6c). Surprisingly, the FPA models display a strong time dependence for classification accuracy for the first 100 ms period after stimulus onset. Moreover, the accuracy largely decreases as code length initially increases. This is because the FPA network has a limited number of discrete attractors, so the encoding space is smaller for the stable patterns than during the transient phase before neural activity has saturated.

LCA models behave more intuitively, with accuracy increasing during the transient phase, and plateauing around 100 ms with better accuracy than the best that FPA models achieve during stimulation. Classification accuracy is dependent on the consistency of the PN responses as well as on the separability between responses to different input ratios. The PN output of LCA models have increasing separability for the first 100 ms since the ratio specificity increases (Figure 6b).

## Discussion

In the present article we presented a neuronal model constructed with a connectivity pattern based on the morphology of the moth MGC, and showed how this model is able to encode ratios between odour concentrations in a binary blend. We aim to contribute to the ongoing discussion about spatial and temporal coding strategies in the insect AL. The two different dynamical regimes FPA and LCA were generated without using any special intrinsic neuron properties (all neurons were first-order linear equations except for the addition of a sigmoidal squashing function), so that as few as possible assumptions were made and we can be sure that they arise as a purely network phenomena, due entirely to the way in which LN inhibitory interconnections were chosen. Using this simple modelling approach, we intend to gain insight into MGC working principles rather than provide an extensive recreation of its physiology in a particular species, because the two types of dynamics explored here may be operative to a certain degree in very different invertebrate nervous systems.

The connectivity of our model was based on morphological studies of the moth, principally *Manduca sexta* [8], [9], [13], [17]–[21], [25] To keep the network patterns as simple as possible, we did not include some features that were described in other insects but not confirmed in the moth, such as the existence of excitatory LNs [26] or feedback from LNs to ORNs [27]. Similarly, the dynamics of the receptors (e.g., [28]) were omitted in order to focus on the ratio encoding capabilities of the MGC circuitry.

From the previous efforts to model different aspects of the AL, only a handful have been specific to the MGC [29]–[36]. Linster *et al.* (1994) describes an oscillatory model, showing that by balancing numbers of inhibitory and excitatory neurons in a network receiving two component input, a system can oscillate when a particular ratio is presented. Linster *et al.* (1996) built a more biologically constrained model including LNs that mediate information to PNs. Again, the balance between inhibitory and excitatory elements is investigated, this time to show that PN response patterns can be made dependent on the input ratio. The model can be tuned such that PNs will display both inhibitory and excitatory influences from the LNs when a particular ratio is presented. As such, model PNs will respond with a pattern of activation, which includes mixed periods of excitation and inhibition, only for input that is close to a particular ratio. The recent article of Zavada *et al.* [36] presents minimalistic feed-forward networks displaying “Winner-Takes-All” type of competition between LNs in the MGC of the moth. These models are based on specific connection strength ratios between ORNs and LNs that allow the recognition of pheromone component ratios across a wide range of concentrations.

In some studies that model the MGC, and those that model the AL for general olfaction, focus has been on generating biologically realistic PN response patterns. Highly-detailed neurons exhibiting bursting behaviour have been used within different neuronal circuits of four-or-less interneurons [29]–[31]. It was shown that these circuits can be designed such that the model PNs display excitation or inhibition depending on which components are present in a simulated pheromone blend input. This replicated some responses observed in the activity of PNs in the Sphinx moth, *Manduca sexta*, when pheromone components were presented [11], [12].

From a theoretical point of view, the issue of excitatory-inhibitory synaptic balance has been addressed in [37], analysing the conditions to have reproducible encoding in “Winnerless Competition” dynamics, which is comparable to LCA. Concerning the FPA dynamics, an AL model of this type has been used for identification of real mixture data using an artificial sensor array to address classification methods and working memory in insect olfactory systems [38], [39].

The existence of a population code in which odours are represented as spatial activation patterns that evolve in time has been extensively described in insects using functional imaging methods, e.g., [9], [40]. On the other hand, both LCA networks and the theoretical framework of “Winnerless Competition” proposed by Laurent's group and collaborators [16], [22], [41]–[44] produce orbits in the PN phase space as a suitable principle by which odour identity and concentration can be represented. In the case of “Winnerless Competition”, the trajectories are defined by sequences of unstable attractors, each corresponding to the transient activation of a specific subset of synchronized PNs. They share the property of being robust against perturbations and, at the same time, very sensitive to the input. There exists considerable evidence that this type of sensory encoding is used in the locust AL [22], [45], and may also constitute a general principle of perceptual representation of multi-dimensional signals widely extended across species and sensory modalities [46], [47]. Our LCA networks show similar sensitivity to the input whilst also being robust to perturbations, although they do not depend upon heteroclinic dynamics.

The facts outlined in the previous paragraph highlight that both spatial and spatiotemporal codes can carry information about odour identity in the insect olfactory system. This illustrates the point of our present modelling effort aimed to address the dynamical behaviours underlying both encoding strategies and compare their ability to encode ratio information. We found that the main advantage of the spatiotemporal code resides in holding more information by its improved specificity between ratios (Figure 6c), and the earlier time in which it reaches the maximum Euclidean distance between the ratio-specific trajectories (legend of Figure 2). The disadvantage is, however, that the potential collision between stimulus and network dynamics can degrade ratio specific information. This is particularly a problem for olfactory coding in moths, since highly dynamic odour plumes occur in the natural environment, generating a temporal structure that is very significant for anemotactic search [23], [24].

We focused on the problem of a potential interference between the stimulus temporal patterns and the network dynamics and found that the LCA encoding of ratios can be indeed disrupted by inter-pulse intervals durations of a range that can occur in pheromonal odour plumes. The simulations show that LCA encoding can seriously fail in certain cases compared with FPA. The interference may arise when frequencies within the natural plume and network dynamics are in the same range. This is not a purely hypothetical issue since frequencies in natural odour plumes have some overlap with those observed in the temporal patterns of PN recordings [23], which also match those found in the dynamics of our LCA model. The real MGC may be able to deal with this problem by having neurons with different time constants so as to ensure the precise encoding in the behaviourally relevant frequency range of stimulus using a LCA scheme. The simulations presented here were done using identical time constants for all the neurons of each type (PNs and LNs). This does not allow a triphasic (inhibition-excitation-inhibition) response pattern as observed in *M. sexta* MGC-PNs [48], except as a random network behaviour. Having a triphasic response pattern would also help to separate the pulses by resetting the responses before coding of the next odour pulse, preventing interference. Another factor that can potentially contribute to avoid interference in biological neural networks is the presence of an offset response to the stimulus that may allow the system to discriminate between blends in spite of collisions between the dynamics of the stimulus and those generated by the network. This type of offset response, described in the locust [49], depends on intrinsic properties of the cells that were not considered in our model. An additional possibility to preserve ratio encoding under complex stimulation patterns would be to rely upon FPA encoding strategies, or to use a combination of FPA and LCA dynamics working in parallel using different neuropils within the MGC.

The biological relevance of the computational simulations presented here can be appreciated in light of Mazor and Laurent's (2005) results in the locust AL. In this study [22], the authors recorded multiple PNs simultaneously while stimulating the antennae with long lasting pulses of odour blends. They found that odour representations can be described as trajectories in PN state space with a transient phase lasting 1–2 seconds, a stable fixed point attractor for about 8 seconds and a final off transient. The optimal stimulus separation occurred during the dynamic phase of the response rather than at the fixed point. Moreover, the period of maximum odour discriminability corresponds to the few hundreds of milliseconds following the stimulus, which is in accordance with behavioural evidence from the reaction time to odours in insects. The dynamical trajectory of the PN vector in the phase space is not only the most informative segment of the response, but it is also responsible for most of the increase in the total activity of the Kenyon cells, that are the targets of the PNs in the mushroom body [22]. The dynamical response that occurs before reaching the fixed point (see Figure 4 in [22]) strongly resembles the LCA dynamics we show in this paper (right panels of Figure 2-A and B), while the fixed points reached some seconds later resemble the FPA framework results (left panels of Figure 2-A and B). Regarding our LCA network, the time in which the trajectories reach maximum Euclidean distance is around 250 ms (legend of Figure 2), and we checked that they reach a fixed point (Figure S1) or a periodic orbit (not shown) after 0.5 to 1 second of simulation. This is similar to Mazor's observations, and differs from the “Winnerless Competition” framework in which a fixed point is never reached (see page 669 in [22]). The late fixed point shown by the LCA network is different from the early fixed point of the FPA network because it includes many LNs active at the same time instead of a single winner. Thus, the difference between both dynamics is not only how fast they can reach a fixed point. As explained above the first 0.5 seconds in which the two dynamics are radically different constitute the most relevant time window from a biological point of view.

In the context of our reductionist modelling approach, many details of MGC morphology and physiology were included as had been observed in the MGC of moths, e.g., the afferent input to the two classes of AL neurons, LNs and PNs, and their interconnections in the glomerular structure [13]. Although it is known that LN dendritic arborization is multiglomerular, more precise connectivity is not known, and our computational simulations test two possible connection arrangements and their respective consequent encoding schemes. Considering the same general architecture and set of synaptic weights, if the LNs' inhibition pattern is symmetric we get FPA dynamics, while an asymmetric connectivity leads to LCA. This is potentially a simple way to explain differences in the dynamics observed in different species that may share a general connectivity pattern in the odour processing networks. More precisely, our results predict that the total LN to LN connectivity should be roughly symmetrical in insects where the spatial coding of odour identity is prominent. Indeed, the odour-specific trajectories in the left panel of Figure 2 appear to follow similarly shaped paths to trajectories of glomerular activity observed in the honeybee [50]. This suggests that the total strength of inhibition between glomeruli should be symmetric. Conversely, in species where temporal patterns seem to be prominent, as the example of the locust mentioned above (e.g., compare the Figure 4 of [22] with the right panels of Figure 2), a more asymmetric inhibition between LNs would be expected. If we consider long term changes in the efficacy of some LN to LN synapses, this line of thinking can also offer possible explanations to learning related modifications in the network dynamics that we plan to address in our forthcoming research.

## Supporting Information

Figure S1 **Fixed point in LCA.** Long lasting LCA simulation showing that the dynamics reaches a fixed point at the end of the first second. The stimulus elapse is shown with a bar under the abscissa. Stimulus ratio was 0.5∶0.5, and LN to LN connection probability 0.25.
(EPS)
